# Exploring the Binding Capacity of Mycelium and Wood-Based Composites for Use in Construction

**DOI:** 10.3390/biomimetics7020078

**Published:** 2022-06-11

**Authors:** Dana Saez, Denis Grizmann, Martin Trautz, Anett Werner

**Affiliations:** 1 Chair of Structures and Structural Design (Trako), Faculty of Architecture, RWTH Aachen University, Schinkelstraße 1, 52062 Aachen, Germany; grizmann@trako.arch.rwth-aachen.de (D.G.); trautz@trako.arch.rwth-aachen.de (M.T.); 2 Group Enzyme Technology, Chair Bioprocess Engineering, Faculty of Mechanical Science and Engineering, Institute of Natural Materials Technology, Technische Universität Dresden, 01069 Dresden, Germany; anett.werner@tu-dresden.de

**Keywords:** binding capacity, bio-adhesives, bio-composites, biomaterials, building biomaterials, fungal mycelium, mechanical performance

## Abstract

Existing research on mycelium-based materials recognizes the binding capacity of fungal hyphae. Fungal hyphae digest and bond to the surface of the substrate, form entangled networks, and enhance the mechanical strength of mycelium-based composites. This investigation was driven by the results of an ongoing project, where we attempt to provide basic concepts for a broad application of a mycelium and chipped wood composite for building components. Simultaneously, we further explore the binding capacity of mycelium and chipped wood composites with a series of experiments involving different mechanical interlocking patterns. Although the matrix material was analyzed on a micro-scale, the samples were developed on a meso-scale to enhance the bonding surface. The meso-scale allows exploring the potential of the bio-based material for use in novel construction systems. The outcome of this study provides a better understanding of the material and geometrical features of mycelium-based building elements.

## 1. Introduction

According to the 2020 Global Status Report for Buildings and Construction published in 2019, the sector moved away from the Paris Agreement goals by causing the highest CO_2_ emissions ever recorded: around 10 Gt CO_2_, or 28% of total global energy-related CO_2_ emissions [1,2]. The increase is mainly related to the carbon-intensive manufacturing processes of building construction materials; therefore, it is crucial to develop novel material strategies to mitigate carbon emissions during the lifecycle of buildings.

Although using renewable raw materials, e.g., wood, presents itself as a logical strategy to withstand CO_2_ emissions, the main problem lies in their production, or, more precisely, their growing time. Looking for alternative renewable materials, recent research has suggested that fast-growing organisms such as Fungal mycelium can be engineered to produce novel construction materials [3,4,5,6]. Mycelium-based composite production is based on the use of lignocellulosic substrates in combination with the natural growth of the vegetative component of the mycelium of filamentous fungi. As filamentous fungi grow, they form hyphae, which result in a close-meshed network and give the resulting material a solid structure. Such composites have many advantages, such as good thermal insulation, low dry density, and sound absorption. These properties make them suitable for use as building materials (e.g., as insulating materials), but they represent a challenge in their load-bearing capacity.

**Figure 1:**
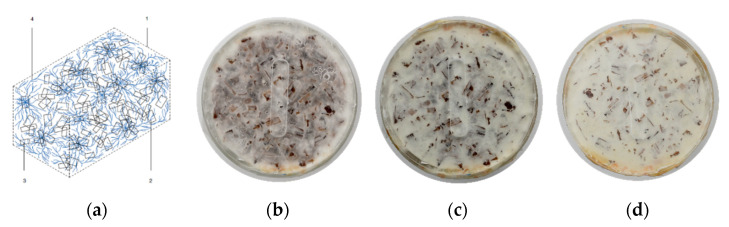
(**a**) The conceptual illustration describes the systematical formation of skeletal hyphae: the dashed line symbolizes the material interfaces (1); the rectangles, substrate consisting of coarse (2) and fine (3) wood chips; and the blue lines, the dense network of mycelial threads (4). The photos show the growth of mycelial cross-linked growth over time (**b**) after two weeks, (**c**) after three weeks, and (**d**) after four weeks.

Our team has been conducting research experiments by developing methods for influencing hyphal growth with the primary objective to provide a mycelium-based composite with particular stability and increased strength [7,8,9,10,11]. In this paper, we describe a manufacturing method of mycelium and wood-based composites where the binding capacity of mycelium plays a crucial role. The fabrication process leads to the fungal mycelium forming predominantly skeletal hyphae at the joint interface of the composite material, which, due to its morphology, leads to increased material strength (Figure 1). This increased strength opens up new application possibilities that go beyond the applications as insulation material or leather substitute [12].

## 2. Materials and Methods

### 2.1. Fungal–Substrate Composition

The findings of this study concern a specific fungal–substrate composition: the fungal mycelium is derived from *Ganoderma lucidum* (GL) and *Pycnoporus sanguineus* (PS), and the substrate is beechwood. Fungi belonging to the genus *Basidiomycetes*, such as GL, *Ganoderma applanatum*, *Trametes hirsuta*, *Trametes versicolor*, or *Fomes fomentarius*, are mostly found in forests and fulfill, among other things, the task of decomposing deadwood. Consequently, we chose timber as the lignocellulosic substrate. Wood consists of approx. 25–30 wt% lignin, 25–30 wt% pentosans (hemicellulose), 40–50 wt% cellulose, and other components such as resinous substances, terpenes fats, fatty acids, proteins, and minerals. Fungi can decompose lignin, hemicellulose, and cellulose into their subunits by releasing enzymes such as cellulases, laccases, amylases, proteases, or lipases into the immediate environment to degrade the substrate. Subsequently, the degradation products are absorbed by the hyphae and used to grow the fungus [10]. The selection of beechwood as the substrate is a consequence of this research team’s previous investigations [2,4,10]. Although the methodology we are about to describe could be transferred to other fungal–substrate compositions, the test results may differ.

### 2.2. Binding-Specific Manufacturing

**Figure 2:**
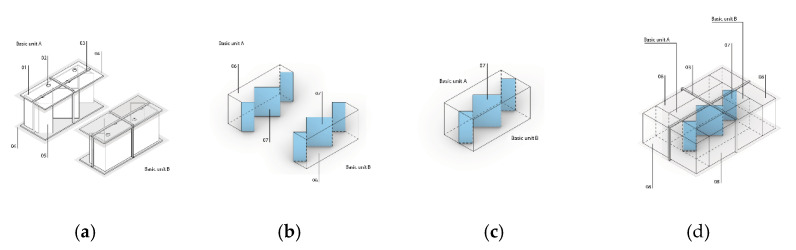
Conceptual illustrations describing the manufacturing process: (**a**) incubation phase II of basic units; (**b**) basic units with binding interface after incubation; (**c**) joining of two basic units; (**d**) incubation phase III of the composite test specimen.

As mentioned before, we seek to provide a mycelium-based composite with a particularly stable and increased strength for use in construction. The manufacturing method proposed consists of the following steps:

(1) Selecting the lignocellulosic substrate.
(2) Inoculating the substrate with fungal spores and fungal mycelium.
(3) Mixing the inoculated substrate so that a homogeneous growth of the mycelium can be achieved.
(4) Incubating the obtained mixture from Step (3) in a first incubation phase for a time between 5 and 7 days, at a temperature ranging from 20 °C to 28 °C, and at humidity ranging from 80% to 95% to achieve the cross-linked growth of the mycelium around the substrate.
(5) Placing the obtained incubated mixture from Step (4) in shaping containers defining the shape of the base unit of the composite material, and incubating the mixture in a second incubation phase for a time between 3 and 10 days, at a temperature ranging from 20 °C to 30 °C, and at humidity ranging from 80% to 95% in order to obtain the cross-linked growth of the mycelium around the substrate.
(6) Obtaining at least two base units of the composite with at least one bonding interface.
(7) Joining at least two basic units through the binding interface and incubating for a time between 10 and 30 days, at a temperature in the range of 15 °C to 30 °C, and at a humidity in the range of 80% to 95% to promote the formation of search hyphae and skeletal hyphae between said basic units and to obtain mycelium and wood-based composite specimens (3rd incubation phase) (Figure 2).

(8) Denaturizing the specimen at a temperature range of 65 °C to 90 °C and obtaining a mycelium-based lignocellulosic composite with a residual moisture content of 10% to 12% by weight based on the total weight of the composite.

Steps (1)–(4) of the manufacturing process are similar to those described in the previous publications of this research team [10]. It is in Step (5) that upon completion of the second incubation phase, at least one base unit of the composite material with at least one binding interface is obtained. A “binding interface” in the present research context refers to the surface to which a different base unit of the composite material can be attached. In the subsequent Step (6), at least two base units of the composite material are provided and subsequently joined so that the binding interfaces of the subunits are joined, as well.

A proper joining configuration is achieved by placing at least two basic units against each other, even though more basic units are able to be attached to form the compound units. Subsequently, the joined basic units are incubated in a third incubation phase (Step (7)). The fungal mycelium grows within the binding interface and couples the two basic units of the composite material.

Finally, the composite material obtained in Step (8) is denaturized to bulk consistency. A mycelium and wood-based lignocellulose composite material is obtained with 10% to 15% residual moisture content.

All manufacturing process steps described above result in the fungal mycelium forming predominantly skeletal hyphae at the joint interface of the composite material, which, due to its morphology, leads to the increased strength of the material in the binding interface.

### 2.3. Planar and Non-Planar Binding Interface

The present research developed a method wherein the binding interface of at least two basic units has a non-planar surface and wherein a factor between 1.2 and 5 enlarges the binding interface compared to that of a planar surface. Whether the surface area of the bonding interfaces is increased by non-planar joint means that the surface of the connection interface has teeth-like interlocking. It could be arranged symmetrically or asymmetrically, which can be connected by the interface of at least two basic units with teeth-like interlocking in the longitudinal section.

The surface of the connecting interface of the base units is enlarged with teeth-like interlocking with jagged (see test specimens Sch2 and Sch3) or rounded protrusions (see test specimens Sch4 and Sch5). These protrusions may be continuous or have at least one short planar section between each jag or curve.

Six different shaping containers were developed to compare, on the one hand, a single test specimen as a single solid unit (Sch0), without binding interface, with at least two base units test samples (Sch1–5). On the other hand, different geometries of the binding interface were developed to compare the influence of the planar and non-planar binding interfaces.

**Figure 3:**
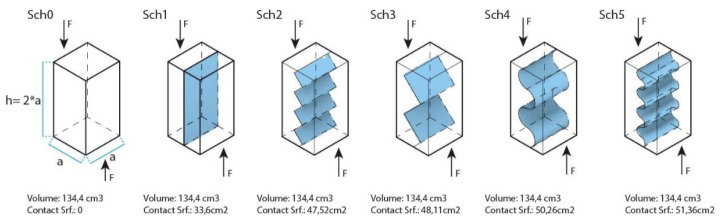
Meso-scale samples for shear tests.

Each container had a 134.4 cm^3^ capacity. Figure 3 and Table 1 describe the exact shapes and sizes of the junction interfaces.

### 2.4. Shear Tests

**Figure 4:**
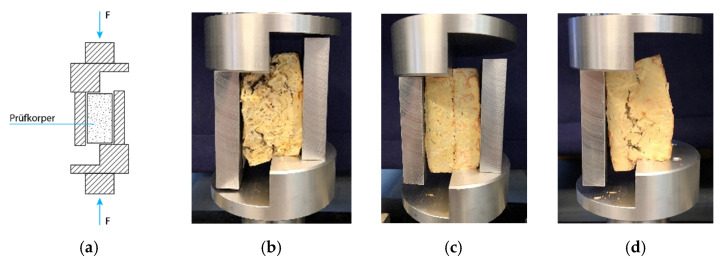
(**a**) Conceptual illustration describing the testing setup. Test specimens being tested under DIN-EN Standards: (**b**) Sch0, specimen with no binding interface; (**c**) Sch1, specimen with planar binding interface; and (**d**) Sch3, specimen with big jagged interface.

Test methods determined the shear strength of the test specimens according to DIN-EN 12090:2013 test standards as illustrated in Figure 4. The test specimens were manufactured as described in Section 2.2 with a consistent filling density of 0.5 g/cm^3^ in a cube-like container (a × a × h). Due to the exploratory character of the testing, two different substrate–fungus combinations were tested: beechwood–GL and beechwood–PS. This variation led to a total amount of 24 Sch test specimens. The shear tests were conducted under laboratory conditions with the corresponding setup, using a Zwick Zmart.Pro testing machine with a testing speed of 3 mm/min. The tests were documented with the help of photographs, and the force deformation curve was digitally recorded with ZickRoell software. The recorded data were subsequently edited and graphically represented in strength diagrams.

## 3. Results

**Figure 5:**
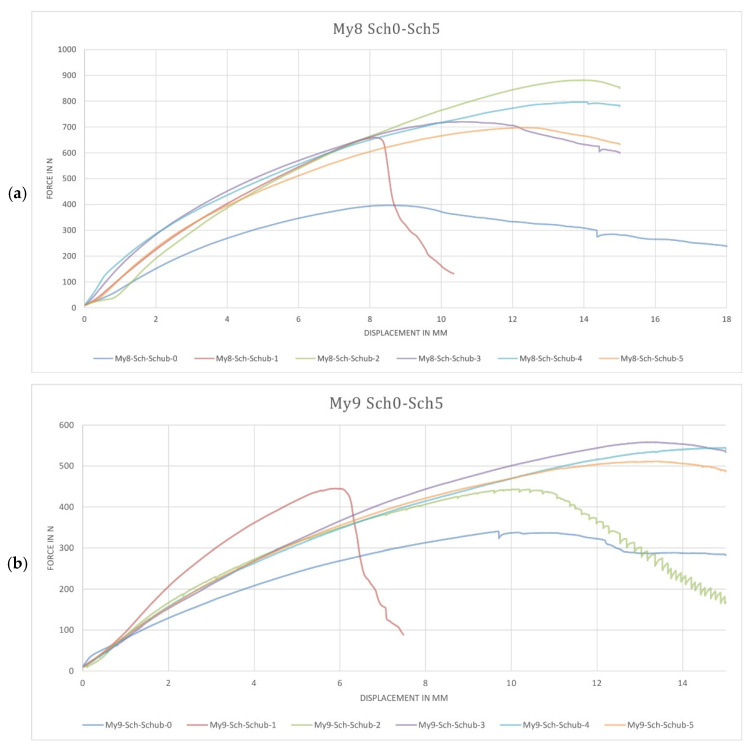
Diagrams showing the recorded force–displacement curves of conducted tests: (**a**) My8—specimen of beechwood–ganoderma lucidum; (**b**) My9—specimen of beechwood–pycnoporus sanguineus.

The obtained material was examined to determine its shear strength. As mentioned before, the tests were carried out on Sch0 to Sch5 test specimens under DIN-EN 12090:2013. The force–displacement curve was recorded for each test, and the shear strength was derived from it (Figure 5).

The first set of analyses presented an apparent variation in fracture behavior. As shown in Figure 5, the blue curves reported significantly less resistance than the rest. Therefore, we can conclude an increase in the material strength of the test specimens developed under binding-specific manufacturing (described in Section 2.2, tests Sch1–5) against those produced under single manufacturing without a binding interface [10]. By repeating the shear tests, this observation was confirmed. These tests were conducted with two different substrate–fungi variations: My8, to beechwood–GL, and My9, to beechwood–PS. Compared with My8, the tests on My9 present significantly less shear resistance.

Non-homogeneous growth on the test specimens may have contributed to the increased variation in the curves in both My8 and My9. The growth variety may result from non-proper environmental conditions in one or more of the cultivation stages during manufacturing.

From the observation of the red curve (Sch1 = planar binding interface) in contrast to the rest of them (Sch0 = no binding interface; Sch2–5 = non-planar binding interface), an influence on the geometry of the inner binding interfaces can be deduced. Consequently, different interface geometries influence the material’s behavior in terms of failure mode, stiffness, and shear strength. The force–displacement curve of specimen Sch1 (red curves) with a planar connection interface, for example, demonstrates an early start of material fracture, which is indicated by a sharp drop in the curve. However, due to the significant curve variation, it has not been possible to determine whether the surface area increase or geometry variation in the binding interface has increased the resistance. Additionally, it would be necessary to repeat the tests with a more significant number of test specimens to determine if the variation between jagged and rounded geometry plays a role in the binding capacity.

The results show that by using the binding ability of the mycelium, it is possible to influence the material properties of composites through the targeted arrangement of joining surfaces with higher stiffness. In this way, direction-dependent material behavior can also be generated. It is also interesting to note that the study of shear strengths reveals that both the interface arrangement and the shear strength are essentially derived from the interface configuration.

## 4. Discussion

The attractiveness of mycelium as a matrix material lies on the one hand in its biological origin, which due to its chemically untreated state, can be easily integrated into the biological material cycle for the circular economy [13]. Due to its manufacturing process, mycelium-based materials have a minimal CO_2_ footprint compared to the vast majority of standard building materials. A wide variety of mycelium-based products have been developed, such as packaging [12] or insulation materials [14]. In general, mycelium-based materials have gained popularity by exploiting the rapid virtual growth of hyphae, which allows the production of conglomerate materials. On the contrary, the positive results on mycelium and wood-based material binding interfaces present this material as an ideal candidate for manufacturing laminar materials. According to the data recorded in Figure 5, the application of binding-specific manufacturing clearly increases the shear strength of the test specimens by at least 50% (average for Sch1 = 0.173 N/mm^2^). Although non-planar binding interfaces present higher shear strength (up to 83%), the results obtained by planar binding interfaces have significant implications for mycelium-based materials. This process could allow the production of multi-laminar elements and could replace oil-based glue materials. Binding-specific manufacturing will doubtless be much scrutinized, but due to the increase in the shear strength, we can conclude that the production process presents advantageous conditions for conglomerates and laminar mycelium and wood-based materials.

With the investigations presented in this paper, it could be assumed that several basic units’ laminar composition can positively influence the load-bearing behavior of mycelium-based materials. However, multiple basic unit composition is not the only factor to consider to achieve building standard requirements. Other factors that positively influence the strength and stiffness properties of mycelium-based materials are mycelium–substrate combinations which could lead to optimal growth or optimal mechanical qualities, and the increase in the material’s density by compression [15]. Further studies that take the latter variables into account will need to be undertaken in combination with binding-specific manufacturing processes.

**Figure 6:**
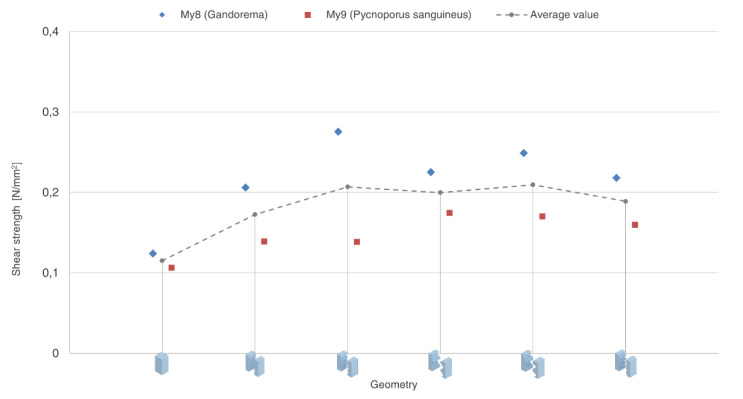
Shear strength to geometry diagram. Test specimen geometries from left to right: Sch0, Sch1, Sch2, Sch3, Sch4, and Sch5.

One of the most important findings of this research is that the geometry of the interfaces can influence fracture behavior. Figure 6 shows how the non-planar binding interfaces show an increased strength. These findings contribute in several ways to a deeper understanding of the binding capacity of mycelial hyphae in combination with wood substrate and provide a basis for further development of mycelium-based material properties.

## 5. Patents

The work reported in this manuscript resulted in the Patent Application No. (DRN) 2021122113010200DE.

## Figures and Tables

**Table 1:** Surface and geometry of the binding interface of the test specimens described in Figure 1.

Test Specimen	Sch0	Sch1	Sch2	Sch3	Sch4	Sch5
Geometry of the binding interface	-	planar	jagged small	jagged big	rounded big	rounded small
Surface of the binding interface (cm^2^)	0	33.6	47.52	48.11	50.26	51.36

## Data Availability

Not applicable.
